# Supplementary material for: Multi‐dataset identification of innovative feature genes and molecular mechanisms in keratoconus
Source: J Cell Mol Med. 2024 Sep 19;28(18):e70079. doi: 10.1111/jcmm.70079 (PMC11412914; doi:10.1111/jcmm.70079)
Supplement: Supplementary file 2 — Material S2. [file JCMM-28-e70079-s002.zip › 7.GSEA analysis/4.MYRF/KEGG_MAPK_SIGNALING_PATHWAY.html]

Details for gene set KEGG\_MAPK\_SIGNALING\_PATHWAY[GSEA]

|  || Dataset | MYRF.group.cls#h\_versus\_l |
| Phenotype | group.cls#h\_versus\_l |
| Upregulated in class | l |
| GeneSet | KEGG\_MAPK\_SIGNALING\_PATHWAY |
| Enrichment Score (ES) | -0.40589902 |
| Normalized Enrichment Score (NES) | -1.6782633 |
| Nominal p-value | 0.002016129 |
| FDR q-value | 0.24179372 |
| FWER p-Value | 0.439 |
Table: GSEA Results Summary

  

Fig 1: Enrichment plot: KEGG\_MAPK\_SIGNALING\_PATHWAY      
 Profile of the Running ES Score & Positions of GeneSet Members on the Rank Ordered List

  

| SYMBOL | TITLE | RANK IN GENE LIST | RANK METRIC SCORE | RUNNING ES | CORE ENRICHMENT || 1 | PLA2G4E | na | 485 | 0.577 | -0.0006 | No |
| 2 | CACNG8 | na | 755 | 0.506 | 0.0033 | No |
| 3 | CACNA1I | na | 1156 | 0.437 | 0.0019 | No |
| 4 | NFATC2 | na | 1281 | 0.423 | 0.0081 | No |
| 5 | PDGFRA | na | 1428 | 0.407 | 0.0134 | No |
| 6 | CACNG5 | na | 1534 | 0.398 | 0.0196 | No |
| 7 | PPP3R2 | na | 2013 | 0.356 | 0.0140 | No |
| 8 | RAC3 | na | 2468 | 0.325 | 0.0084 | No |
| 9 | FGF8 | na | 2858 | 0.303 | 0.0042 | No |
| 10 | CHP2 | na | 3253 | 0.283 | -0.0007 | No |
| 11 | ELK1 | na | 3767 | 0.262 | -0.0095 | No |
| 12 | DUSP9 | na | 4778 | 0.229 | -0.0334 | No |
| 13 | RPS6KA2 | na | 4975 | 0.223 | -0.0339 | No |
| 14 | NTRK1 | na | 5375 | 0.211 | -0.0406 | No |
| 15 | FGF18 | na | 5499 | 0.207 | -0.0393 | No |
| 16 | CACNB1 | na | 5538 | 0.206 | -0.0356 | No |
| 17 | FGF16 | na | 5776 | 0.199 | -0.0379 | No |
| 18 | SOS1 | na | 6706 | 0.172 | -0.0608 | No |
| 19 | HSPA8 | na | 7113 | 0.161 | -0.0688 | No |
| 20 | MECOM | na | 7274 | 0.157 | -0.0698 | No |
| 21 | TAOK2 | na | 7290 | 0.156 | -0.0666 | No |
| 22 | RASGRP2 | na | 7635 | 0.148 | -0.0732 | No |
| 23 | FGF5 | na | 7739 | 0.145 | -0.0728 | No |
| 24 | PLA2G3 | na | 8083 | 0.136 | -0.0796 | No |
| 25 | FGF3 | na | 8177 | 0.134 | -0.0792 | No |
| 26 | BRAF | na | 8198 | 0.133 | -0.0766 | No |
| 27 | CACNA1B | na | 8324 | 0.131 | -0.0772 | No |
| 28 | MAPK9 | na | 8409 | 0.129 | -0.0766 | No |
| 29 | MAPK12 | na | 8572 | 0.125 | -0.0784 | No |
| 30 | RPS6KA6 | na | 8598 | 0.125 | -0.0763 | No |
| 31 | FGFR4 | na | 8772 | 0.121 | -0.0785 | No |
| 32 | FGF12 | na | 8823 | 0.120 | -0.0771 | No |
| 33 | CASP3 | na | 8824 | 0.120 | -0.0743 | No |
| 34 | ARRB1 | na | 9053 | 0.115 | -0.0783 | No |
| 35 | KRAS | na | 9371 | 0.108 | -0.0849 | No |
| 36 | RASGRF1 | na | 9396 | 0.108 | -0.0831 | No |
| 37 | RASA1 | na | 9714 | 0.101 | -0.0900 | No |
| 38 | MAP3K4 | na | 9809 | 0.099 | -0.0904 | No |
| 39 | PTPRR | na | 9918 | 0.097 | -0.0913 | No |
| 40 | NF1 | na | 10055 | 0.095 | -0.0930 | No |
| 41 | PLA2G12B | na | 10192 | 0.093 | -0.0948 | No |
| 42 | AKT2 | na | 10210 | 0.092 | -0.0931 | No |
| 43 | FGF17 | na | 10236 | 0.092 | -0.0917 | No |
| 44 | FGF13 | na | 10299 | 0.091 | -0.0914 | No |
| 45 | FLNB | na | 10346 | 0.090 | -0.0907 | No |
| 46 | MAPK11 | na | 10606 | 0.085 | -0.0962 | No |
| 47 | GADD45G | na | 10627 | 0.084 | -0.0948 | No |
| 48 | CACNB2 | na | 10834 | 0.081 | -0.0989 | No |
| 49 | MAPK13 | na | 10951 | 0.079 | -0.1004 | No |
| 50 | PLA2G2D | na | 11306 | 0.073 | -0.1090 | No |
| 51 | CACNA2D3 | na | 11412 | 0.072 | -0.1104 | No |
| 52 | MAP3K6 | na | 11468 | 0.071 | -0.1103 | No |
| 53 | DUSP3 | na | 11609 | 0.069 | -0.1128 | No |
| 54 | PRKX | na | 11663 | 0.068 | -0.1127 | No |
| 55 | PPM1B | na | 11813 | 0.066 | -0.1155 | No |
| 56 | CACNA1C | na | 11956 | 0.064 | -0.1181 | No |
| 57 | MAPK14 | na | 11965 | 0.064 | -0.1169 | No |
| 58 | ELK4 | na | 12068 | 0.063 | -0.1184 | No |
| 59 | HSPA2 | na | 12165 | 0.061 | -0.1198 | No |
| 60 | MAP3K3 | na | 12309 | 0.059 | -0.1225 | No |
| 61 | CD14 | na | 12315 | 0.059 | -0.1213 | No |
| 62 | RPS6KA3 | na | 12372 | 0.058 | -0.1216 | No |
| 63 | MAPK10 | na | 12440 | 0.057 | -0.1222 | No |
| 64 | SOS2 | na | 12783 | 0.053 | -0.1309 | No |
| 65 | FGF20 | na | 12795 | 0.053 | -0.1300 | No |
| 66 | TP53 | na | 12977 | 0.051 | -0.1340 | No |
| 67 | IKBKB | na | 12994 | 0.050 | -0.1333 | No |
| 68 | TAOK1 | na | 13183 | 0.048 | -0.1377 | No |
| 69 | MAP2K4 | na | 13264 | 0.047 | -0.1389 | No |
| 70 | TRAF2 | na | 13353 | 0.046 | -0.1404 | No |
| 71 | CACNA1D | na | 13802 | 0.040 | -0.1525 | No |
| 72 | MAP3K1 | na | 13876 | 0.039 | -0.1537 | No |
| 73 | FGF9 | na | 13888 | 0.039 | -0.1531 | No |
| 74 | PDGFA | na | 13993 | 0.038 | -0.1552 | No |
| 75 | MAP2K2 | na | 14012 | 0.038 | -0.1548 | No |
| 76 | RPS6KA5 | na | 14158 | 0.036 | -0.1582 | No |
| 77 | PLA2G12A | na | 14161 | 0.036 | -0.1574 | No |
| 78 | PRKCA | na | 14202 | 0.036 | -0.1578 | No |
| 79 | CHUK | na | 14348 | 0.034 | -0.1612 | No |
| 80 | MAPK3 | na | 14382 | 0.034 | -0.1614 | No |
| 81 | FGFR3 | na | 14422 | 0.033 | -0.1617 | No |
| 82 | PLA2G2C | na | 14507 | 0.032 | -0.1634 | No |
| 83 | RASGRF2 | na | 14631 | 0.031 | -0.1662 | No |
| 84 | TAOK3 | na | 14720 | 0.030 | -0.1681 | No |
| 85 | MKNK2 | na | 15346 | 0.024 | -0.1856 | No |
| 86 | FGF7 | na | 15518 | 0.023 | -0.1901 | No |
| 87 | RPS6KA4 | na | 15544 | 0.022 | -0.1903 | No |
| 88 | MAPK7 | na | 15567 | 0.022 | -0.1904 | No |
| 89 | PLA2G10 | na | 15631 | 0.022 | -0.1917 | No |
| 90 | DAXX | na | 15690 | 0.021 | -0.1929 | No |
| 91 | MAPKAPK3 | na | 15849 | 0.019 | -0.1971 | No |
| 92 | PRKCG | na | 15973 | 0.018 | -0.2002 | No |
| 93 | NLK | na | 16181 | 0.016 | -0.2059 | No |
| 94 | CACNG7 | na | 16278 | 0.015 | -0.2083 | No |
| 95 | FASLG | na | 16299 | 0.015 | -0.2085 | No |
| 96 | JUN | na | 16671 | 0.011 | -0.2190 | No |
| 97 | HSPA1B | na | 16673 | 0.011 | -0.2188 | No |
| 98 | MKNK1 | na | 16817 | 0.010 | -0.2227 | No |
| 99 | EGFR | na | 17031 | 0.008 | -0.2287 | No |
| 100 | ARRB2 | na | 17178 | 0.007 | -0.2328 | No |
| 101 | RAC2 | na | 17259 | 0.006 | -0.2349 | No |
| 102 | RAP1A | na | 17348 | 0.006 | -0.2374 | No |
| 103 | MAX | na | 17507 | 0.004 | -0.2419 | No |
| 104 | PPP3R1 | na | 17799 | 0.001 | -0.2503 | No |
| 105 | PLA2G6 | na | 17909 | 0.000 | -0.2534 | No |
| 106 | CACNG1 | na | 17979 | -0.000 | -0.2554 | No |
| 107 | AKT1 | na | 18101 | -0.001 | -0.2589 | No |
| 108 | PLA2G2F | na | 18318 | -0.004 | -0.2651 | No |
| 109 | RAPGEF2 | na | 18411 | -0.004 | -0.2676 | No |
| 110 | STK4 | na | 18494 | -0.005 | -0.2699 | No |
| 111 | FGF19 | na | 18545 | -0.006 | -0.2712 | No |
| 112 | HSPA1A | na | 18580 | -0.006 | -0.2721 | No |
| 113 | MAPK8 | na | 18612 | -0.006 | -0.2728 | No |
| 114 | CACNG2 | na | 18631 | -0.007 | -0.2732 | No |
| 115 | FGF10 | na | 18767 | -0.008 | -0.2769 | No |
| 116 | PLA2G2E | na | 18859 | -0.009 | -0.2794 | No |
| 117 | RAP1B | na | 18871 | -0.009 | -0.2795 | No |
| 118 | EGF | na | 19142 | -0.012 | -0.2870 | No |
| 119 | CHP1 | na | 19165 | -0.012 | -0.2874 | No |
| 120 | STK3 | na | 19181 | -0.013 | -0.2875 | No |
| 121 | MAP3K11 | na | 19187 | -0.013 | -0.2874 | No |
| 122 | TAB1 | na | 19282 | -0.014 | -0.2898 | No |
| 123 | MAP2K7 | na | 19405 | -0.015 | -0.2930 | No |
| 124 | PRKACA | na | 19584 | -0.017 | -0.2977 | No |
| 125 | FGF11 | na | 19615 | -0.017 | -0.2982 | No |
| 126 | MOS | na | 20086 | -0.023 | -0.3113 | No |
| 127 | MAPK8IP3 | na | 20223 | -0.024 | -0.3147 | No |
| 128 | MAPK1 | na | 20406 | -0.027 | -0.3194 | No |
| 129 | FOS | na | 20589 | -0.029 | -0.3240 | No |
| 130 | FGF14 | na | 20664 | -0.030 | -0.3254 | No |
| 131 | JMJD7-PLA2G4B | na | 20701 | -0.030 | -0.3258 | No |
| 132 | HSPB1 | na | 20810 | -0.031 | -0.3282 | No |
| 133 | PTPN5 | na | 20827 | -0.031 | -0.3279 | No |
| 134 | FGFR2 | na | 20983 | -0.033 | -0.3316 | No |
| 135 | MAP2K5 | na | 21130 | -0.035 | -0.3351 | No |
| 136 | ATF4 | na | 21458 | -0.039 | -0.3436 | No |
| 137 | CACNA1A | na | 21608 | -0.041 | -0.3470 | No |
| 138 | IKBKG | na | 21687 | -0.042 | -0.3483 | No |
| 139 | RAF1 | na | 21720 | -0.042 | -0.3482 | No |
| 140 | PLA2G4A | na | 21803 | -0.043 | -0.3496 | No |
| 141 | HRAS | na | 21856 | -0.044 | -0.3501 | No |
| 142 | RAC1 | na | 21936 | -0.045 | -0.3513 | No |
| 143 | TGFB2 | na | 22177 | -0.048 | -0.3572 | No |
| 144 | PPP3CA | na | 22219 | -0.049 | -0.3572 | No |
| 145 | ECSIT | na | 22232 | -0.049 | -0.3565 | No |
| 146 | MAPKAPK5 | na | 22388 | -0.051 | -0.3598 | No |
| 147 | BDNF | na | 22475 | -0.052 | -0.3611 | No |
| 148 | TAB2 | na | 22545 | -0.053 | -0.3618 | No |
| 149 | PPP3CB | na | 22763 | -0.056 | -0.3668 | No |
| 150 | PPM1A | na | 23033 | -0.060 | -0.3732 | No |
| 151 | MAP4K2 | na | 23049 | -0.060 | -0.3723 | No |
| 152 | TNFRSF1A | na | 23281 | -0.064 | -0.3775 | No |
| 153 | CDC42 | na | 23297 | -0.064 | -0.3764 | No |
| 154 | HSPA1L | na | 23422 | -0.066 | -0.3785 | No |
| 155 | DUSP8 | na | 23425 | -0.066 | -0.3770 | No |
| 156 | TRAF6 | na | 23526 | -0.068 | -0.3783 | No |
| 157 | MAP2K3 | na | 23613 | -0.069 | -0.3792 | No |
| 158 | MAP3K2 | na | 23997 | -0.076 | -0.3886 | No |
| 159 | GRB2 | na | 24215 | -0.079 | -0.3930 | No |
| 160 | DUSP16 | na | 24273 | -0.080 | -0.3928 | No |
| 161 | PAK2 | na | 24412 | -0.082 | -0.3949 | No |
| 162 | NTF3 | na | 24484 | -0.084 | -0.3950 | No |
| 163 | FGF22 | na | 24497 | -0.084 | -0.3934 | No |
| 164 | RASGRP4 | na | 24628 | -0.087 | -0.3951 | No |
| 165 | MAP3K13 | na | 24700 | -0.088 | -0.3952 | No |
| 166 | MAP3K7 | na | 24724 | -0.088 | -0.3938 | No |
| 167 | TGFBR2 | na | 24993 | -0.093 | -0.3994 | No |
| 168 | MAPK8IP1 | na | 25049 | -0.094 | -0.3988 | No |
| 169 | RASA2 | na | 25050 | -0.094 | -0.3966 | No |
| 170 | GNA12 | na | 25107 | -0.095 | -0.3960 | No |
| 171 | GNG12 | na | 25293 | -0.099 | -0.3991 | No |
| 172 | CRK | na | 25398 | -0.101 | -0.3997 | No |
| 173 | PPP5C | na | 25464 | -0.102 | -0.3993 | No |
| 174 | TGFBR1 | na | 25528 | -0.103 | -0.3987 | No |
| 175 | MAP4K4 | na | 25691 | -0.106 | -0.4009 | No |
| 176 | PRKCB | na | 25770 | -0.108 | -0.4007 | No |
| 177 | MAPK8IP2 | na | 25891 | -0.110 | -0.4016 | No |
| 178 | LAMTOR3 | na | 25909 | -0.111 | -0.3995 | No |
| 179 | RELB | na | 26126 | -0.116 | -0.4031 | No |
| 180 | CACNB3 | na | 26225 | -0.118 | -0.4032 | Yes |
| 181 | FGF21 | na | 26242 | -0.118 | -0.4009 | Yes |
| 182 | PAK1 | na | 26325 | -0.120 | -0.4005 | Yes |
| 183 | MAPKAPK2 | na | 26341 | -0.120 | -0.3981 | Yes |
| 184 | RRAS2 | na | 26466 | -0.122 | -0.3989 | Yes |
| 185 | PDGFB | na | 26504 | -0.123 | -0.3971 | Yes |
| 186 | DUSP14 | na | 26633 | -0.126 | -0.3978 | Yes |
| 187 | CACNA1H | na | 26649 | -0.127 | -0.3953 | Yes |
| 188 | FGF6 | na | 27010 | -0.135 | -0.4026 | Yes |
| 189 | MAP3K5 | na | 27057 | -0.137 | -0.4008 | Yes |
| 190 | NGF | na | 27092 | -0.137 | -0.3986 | Yes |
| 191 | PDGFRB | na | 27235 | -0.141 | -0.3994 | Yes |
| 192 | DUSP1 | na | 27292 | -0.142 | -0.3977 | Yes |
| 193 | RRAS | na | 27376 | -0.144 | -0.3968 | Yes |
| 194 | FLNC | na | 27638 | -0.151 | -0.4008 | Yes |
| 195 | MYC | na | 27707 | -0.153 | -0.3992 | Yes |
| 196 | IL1R2 | na | 27718 | -0.153 | -0.3960 | Yes |
| 197 | GADD45B | na | 27736 | -0.154 | -0.3929 | Yes |
| 198 | FGF2 | na | 27758 | -0.154 | -0.3899 | Yes |
| 199 | FGF1 | na | 27813 | -0.156 | -0.3878 | Yes |
| 200 | FAS | na | 28092 | -0.163 | -0.3921 | Yes |
| 201 | NFKB1 | na | 28241 | -0.168 | -0.3925 | Yes |
| 202 | ATF2 | na | 28284 | -0.169 | -0.3898 | Yes |
| 203 | PPP3CC | na | 28351 | -0.171 | -0.3877 | Yes |
| 204 | CDC25B | na | 28392 | -0.172 | -0.3848 | Yes |
| 205 | MAP4K3 | na | 28438 | -0.173 | -0.3821 | Yes |
| 206 | CACNA1G | na | 28472 | -0.174 | -0.3790 | Yes |
| 207 | DUSP6 | na | 28632 | -0.179 | -0.3795 | Yes |
| 208 | PTPN7 | na | 28776 | -0.184 | -0.3793 | Yes |
| 209 | STMN1 | na | 28836 | -0.186 | -0.3767 | Yes |
| 210 | CACNA1F | na | 28862 | -0.187 | -0.3731 | Yes |
| 211 | IL1R1 | na | 28913 | -0.188 | -0.3701 | Yes |
| 212 | CRKL | na | 29092 | -0.194 | -0.3708 | Yes |
| 213 | RPS6KA1 | na | 29168 | -0.196 | -0.3684 | Yes |
| 214 | JUND | na | 29242 | -0.198 | -0.3659 | Yes |
| 215 | MAP4K1 | na | 29278 | -0.199 | -0.3623 | Yes |
| 216 | CACNG6 | na | 29465 | -0.205 | -0.3629 | Yes |
| 217 | NTF4 | na | 29548 | -0.208 | -0.3604 | Yes |
| 218 | NRAS | na | 30019 | -0.224 | -0.3689 | Yes |
| 219 | FGF23 | na | 30092 | -0.227 | -0.3657 | Yes |
| 220 | MAP2K1 | na | 30150 | -0.229 | -0.3620 | Yes |
| 221 | MAP2K6 | na | 30203 | -0.231 | -0.3581 | Yes |
| 222 | PLA2G5 | na | 30433 | -0.240 | -0.3592 | Yes |
| 223 | FLNA | na | 30801 | -0.254 | -0.3639 | Yes |
| 224 | NFKB2 | na | 31039 | -0.266 | -0.3646 | Yes |
| 225 | RELA | na | 31135 | -0.271 | -0.3611 | Yes |
| 226 | PRKACG | na | 31385 | -0.285 | -0.3616 | Yes |
| 227 | RASGRP1 | na | 31683 | -0.300 | -0.3633 | Yes |
| 228 | CACNG4 | na | 31907 | -0.312 | -0.3625 | Yes |
| 229 | CACNA2D2 | na | 32005 | -0.317 | -0.3579 | Yes |
| 230 | NR4A1 | na | 32261 | -0.334 | -0.3575 | Yes |
| 231 | CACNA2D4 | na | 32488 | -0.350 | -0.3559 | Yes |
| 232 | DUSP7 | na | 32517 | -0.352 | -0.3486 | Yes |
| 233 | MAP3K14 | na | 32615 | -0.359 | -0.3430 | Yes |
| 234 | GADD45A | na | 32736 | -0.368 | -0.3380 | Yes |
| 235 | TGFB1 | na | 32868 | -0.377 | -0.3330 | Yes |
| 236 | NFATC4 | na | 32887 | -0.379 | -0.3247 | Yes |
| 237 | IL1A | na | 32896 | -0.380 | -0.3160 | Yes |
| 238 | PLA2G2A | na | 33011 | -0.388 | -0.3103 | Yes |
| 239 | TGFB3 | na | 33024 | -0.389 | -0.3016 | Yes |
| 240 | NTRK2 | na | 33066 | -0.393 | -0.2936 | Yes |
| 241 | SRF | na | 33126 | -0.399 | -0.2861 | Yes |
| 242 | HSPA6 | na | 33174 | -0.404 | -0.2780 | Yes |
| 243 | DUSP4 | na | 33195 | -0.406 | -0.2691 | Yes |
| 244 | CACNA1S | na | 33196 | -0.406 | -0.2597 | Yes |
| 245 | DDIT3 | na | 33385 | -0.429 | -0.2551 | Yes |
| 246 | PRKACB | na | 33438 | -0.435 | -0.2465 | Yes |
| 247 | MAPT | na | 33499 | -0.443 | -0.2380 | Yes |
| 248 | DUSP2 | na | 33543 | -0.450 | -0.2287 | Yes |
| 249 | CACNA1E | na | 33690 | -0.468 | -0.2221 | Yes |
| 250 | MAP3K12 | na | 34181 | -0.567 | -0.2231 | Yes |
| 251 | CACNB4 | na | 34188 | -0.569 | -0.2100 | Yes |
| 252 | CACNG3 | na | 34232 | -0.580 | -0.1978 | Yes |
| 253 | IL1B | na | 34248 | -0.586 | -0.1846 | Yes |
| 254 | TNF | na | 34328 | -0.609 | -0.1727 | Yes |
| 255 | FGF4 | na | 34351 | -0.616 | -0.1590 | Yes |
| 256 | MRAS | na | 34392 | -0.631 | -0.1455 | Yes |
| 257 | MEF2C | na | 34404 | -0.636 | -0.1310 | Yes |
| 258 | MAP3K8 | na | 34409 | -0.639 | -0.1162 | Yes |
| 259 | RASGRP3 | na | 34457 | -0.659 | -0.1022 | Yes |
| 260 | DUSP10 | na | 34533 | -0.694 | -0.0883 | Yes |
| 261 | PLA2G1B | na | 34558 | -0.712 | -0.0724 | Yes |
| 262 | CACNA2D1 | na | 34582 | -0.726 | -0.0561 | Yes |
| 263 | DUSP5 | na | 34594 | -0.733 | -0.0394 | Yes |
| 264 | FGFR1 | na | 34693 | -0.924 | -0.0207 | Yes |
| 265 | AKT3 | na | 34696 | -0.932 | 0.0009 | Yes |
Table: GSEA details [plain text format]

  

Fig 2: KEGG\_MAPK\_SIGNALING\_PATHWAY      
 Blue-Pink O' Gram in the Space of the Analyzed GeneSet

  

Fig 3: KEGG\_MAPK\_SIGNALING\_PATHWAY: Random ES distribution      
 Gene set null distribution of ES for **KEGG\_MAPK\_SIGNALING\_PATHWAY**
